# Smoking during pregnancy and children’s emotional and behavioural trajectories

**DOI:** 10.1093/eurpub/ckac129.669

**Published:** 2022-10-25

**Authors:** M Fekom, K Bonello, R Gomajee, G Ibanez, S Martin, K Keyes, A Nakamura, J Lepeule, K Strandberg-Larsen, M Melchior

**Affiliations:** 1 General Practice, Sorbonne University, School of Medicine, Paris, France; 2 Social Epidemiology, Sorbonne Université, INSERM, Institut Pierre, Paris, France; 3 Epidemiology, Mailman School of Public Health, Columbia University, New York, USA; 4 Institute for Advanced Biosciences, Université Grenoble Alpes, INSERM, CNRS, Grenoble, France; 5 Epidemiology, Public Health, University of Copenhagen, Copenhagen, Denmark

## Abstract

**Background:**

The nature of the relationship between maternal tobacco smoking during pregnancy and the occurrence of children's behavioural problems is still a matter of controversy. We tested this association using data collected among a sample of children followed from pregnancy to early adolescence (age 11.5 years), accounting for multiple parents’, children's and family characteristics.

**Methods:**

Data come from 1424 mother-child pairs participating in the EDEN mother-child cohort set up in France. Using repeated measures (3, 5.5, 8 and 11.5 years) of the mother-reported Strengths and Difficulties Questionnaire, we estimated trajectories of children's emotional and behavioural difficulties. Two aspects of maternal smoking were studied: the timing and the level of use (cigarettes/day) during the first trimester of pregnancy. Multinomial regression models controlled for confounding factors including maternal mental health and socioeconomic characteristics using propensity scores with the overlap weighting technique.

**Results:**

Contrary to bivariate analyses, in propensity score-controlled regression models, maternal smoking throughout pregnancy was no longer significantly associated with offspring emotional or behavioural difficulties. Maternal heavy smoking (≥10cigarettes/day) remained significantly associated with intermediate levels of overall emotional and behavioural difficulties (OR 1.64, 95%CI 1.04-2.58) and conduct problems (OR 3.05 95%CI 1.22-7.61), as well as with high levels of conduct problems symptoms (OR 2.82 95%CI 0.88-9.06) - although the latter did not reach statistical significance.

**Conclusions:**

The association between maternal smoking in pregnancy and offspring emotional and behavioural difficulties appears to be largely explained by women's other characteristics. However, maternal heavy smoking appears to be related to offspring behavioural difficulties beyond the role of confounding characteristics.

**Key messages:**

• The association between maternal smoking in pregnancy and offspring emotional and behavioural difficulties seem largely explained by the family's socio-demographic and behavioural characteristics.

• Maternal heavy smoking appears to be related to offspring behavioural difficulties beyond the role of confounding characteristics.

